# Comparison of stereopsis and foveal microstructure after internal limiting membrane peeling and inverted internal limiting membrane flap techniques in patients with macular hole

**DOI:** 10.1371/journal.pone.0297134

**Published:** 2024-02-09

**Authors:** Shohei Morikawa, Fumiki Okamoto, Tomoya Murakami, Yoshimi Sugiura, Tetsuro Oshika

**Affiliations:** Department of Ophthalmology, Faculty of Medicine, University of Tsukuba, Tsukuba, Ibaraki, Japan; St. Marianna University School of Medicine, JAPAN

## Abstract

**Purpose:**

To compare stereopsis and foveal microstructure after internal limiting membrane peeling and inverted internal limiting membrane flap technique in patients with macular hole.

**Design:**

Retrospective observational study.

**Methods:**

Sixty-six patients with macular hole were included, of whom 41 underwent 25-gauge pars-plana vitrectomy with complete internal limiting membrane peeling (Peeling group) and 25 with the inverted flap technique (Inverted group). We evaluated stereopsis using the Titmus Stereo Test and the TNO stereo test, best-corrected visual acuity, macular hole closure rate, and foveal microstructure with optical coherence tomography before and at 3, 6, and 12 months after surgery.

**Main outcome measures:**

Stereopsis and foveal microstructure.

**Results:**

Preoperatively, no difference was observed in the base and minimum diameters of macular hole, Titmus Stereo Test score, TNO stereo test score, and best-corrected visual acuity between the Peeling and Inverted groups. The macular hole closure rate in the Peeling and Inverted groups were 97.6% and 100%, respectively, with no significant difference between groups. At 12 months postoperatively, Titmus Stereo Test score (2.1 ± 0.4 in the peeling and 2.2 ± 0.4 in the inverted groups), TNO stereo test score (2.3 ± 0.4 and 2.2± 0.5), and best-corrected visual acuity (0.20 ± 0.18 and 0.24 ± 0.25) were not significantly different between groups (p = 0.596, 0.332, respectively). The defect of the external limiting membrane was more common in the Inverted group than in the Peeling group at 6 months after surgery (5.4 vs. 28.0%; p < 0.05). No statistically significant inter-group differences were noted in the ellipsoid zone defect ratio throughout the follow-up period.

**Conclusions:**

There was no difference in postoperative stereopsis nor foveal microstructure between the internal limiting membrane peeling group and the inverted group in patients with macular hole.

## Introduction

An idiopathic full-thickness macular hole (MH) is a vitreoretinal interface disease with a retinal defect in the center of the macula and results in the loss of various visual functions such as visual acuity, aniseikonia, metamorphopsia, and stereopsis [1–7]. Vitrectomy is the one of means to treat MH and has contributed to improved visual acuity [8–11]. Stereopsis is the most advanced visual function that enables depth of field recognition based on the image disparity formed by the two eyes. Stereopsis was found to be impaired, affecting quality of life, in patients with unilateral retinal diseases, including MH [4–7], epiretinal membrane [12, 13], and branch retinal vein occlusion [14–16]. In addition, stereopsis in MH was associated with retinal microstructure, including defect length of the external limiting membrane (ELM) and interdigitation zone [5, 6].

Various surgical techniques have been reported to achieve anatomic improvement for MH, including vitrectomy with gas tamponade, internal limiting membrane (ILM) peeling, and inverted ILM flap technique [8–11]. Currently, the two most commonly used surgical techniques are vitrectomy with ILM peeling and inverted ILM flap technique for MH. Previous studies reported that the inverted ILM flap technique might be better than ILM peeling for the treatment of large MH [11, 17–21]. In patients undergoing surgery for small and medium-sized MH, postoperative best-corrected visual acuity (BCVA) was similar for the ILM peeling and inverted ILM flap technique [22, 23]. The treatment with vitrectomy with ILM peeling improved stereopsis in patients with MH [7], whereas whether vitrectomy with inverted ILM flap technique could restore stereopsis is unknown.

To the best of our knowledge, no study reveals the best procedure for stereopsis in patients with MH. This study aimed to compare the stereopsis and anatomical outcomes of the ILM peeling and the inverted ILM flap technique in the treatment of MH and to evaluate reconstructive anatomical changes in foveal microstructure using Optical Coherence Tomography (OCT).

## Materials and methods

A retrospective chart review of 66 eyes of 66 patients was conducted, consisting of 29 males and 37 females averaging 65.5 ± 5.7 years of age (mean ± standard deviations) (range: 55–83 years) who had undergone 25-gauge pars-plana vitrectomy with complete ILM peeling or inverted ILM flap technique for unilateral idiopathic MH at Tsukuba University Hospital. The exclusion criteria included high myopia (spherical equivalent ≤ -6D), presence of staphyloma, secondary MH (proliferative diabetic retinopathy, macular edema, retinal detachment, ocular trauma, and uveitis), recurrent macular hole, history of vitreous surgery or scleral buckling. Stereopsis was impaired by anisometropia over 2 diopters, thus that were excluded before and after surgery. This study was conducted in accordance with the tenets of the Declaration of Helsinki, and the Institutional Review Board of the University of Tsukuba approval was obtained (approval number: R02-40). All participants were informed of their rights, and they provided written informed consent before undergoing the study procedures.

All surgeries were performed by one experienced vitreoretinal surgeon (F.O.). All patients underwent 25-gauge pars plana vitrectomy with the Constellation Vision System (Alcon Laboratories, Inc). Patients with cataracts with nuclear sclerosis grade 2 or cortical opacity underwent simultaneous cataract surgery according to LOCS III classification [24]. After under sub-Tenon local anesthesia, three-port transconjunctival sclerostomy was made by oblique sclerotome. When required, triamcinolone acetonide was used to secure posterior vitreous detachment and remove the posterior hyaloid membrane after core vitrectomy. If a retinal break was found, retinal photocoagulation was performed around it. We injected 0.2 mL of 0.025% brilliant blue G solution intravitreally with a slow stream directed toward the posterior pole of the eye to stain the ILM.

The ILM was grasped with ILM forceps and peeled off centripetally, about two disk diameters around the MH in ILM peeling procedure. The inverted ILM flap technique was performed according to the ordinal method of report by Michalewska et al. [11]. During ILM peeling around the MH, the ILM flap was not completely removed from the retina, but a remnant was left attached to the edges of the MH. The ILM flap was single layer for 1.5 of MH and placed without embedding. Fluid-air exchange was performed in all cases. All patients with MH were kept in the prone position for at least 1 day postoperatively. The initial 41 cases were consecutively treated with ILM peeling, followed by the subsequent 25 cases with consecutive ILM inverted procedures. The choice of surgical technique was not based on the size or shape of the macular hole. Surgery was considered successful when a complete MH closure (defined as the absence of a neurosensory defect over the retina) was achieved and the closure status was checked within 3 months postoperatively.

**Fig 1:**
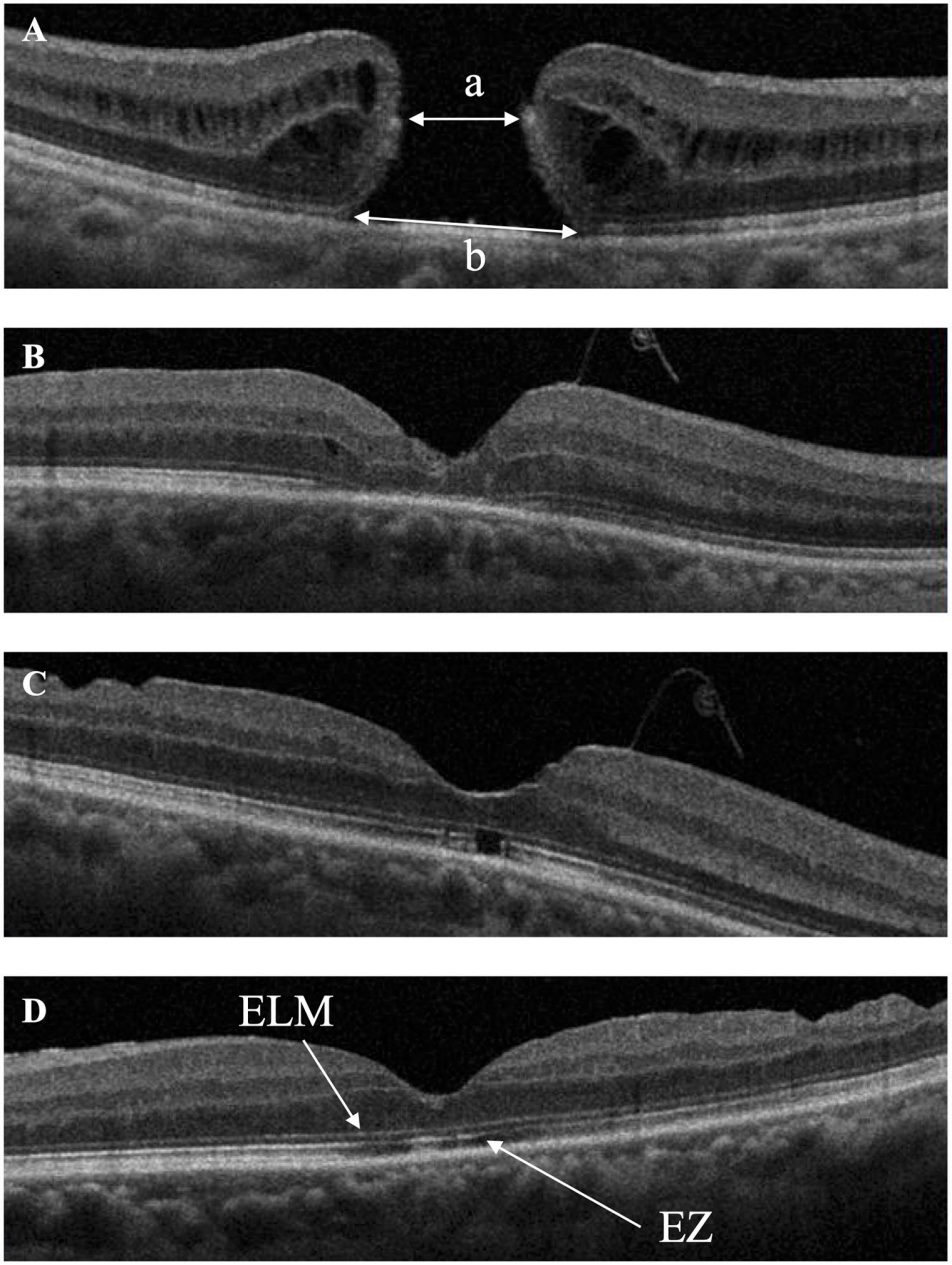
Macular hole (MH) parameters based on optical coherence tomography images before and after surgery. A: We measured the minimum diameters of MH (a) and base diameters of MH (b) before surgery. We evaluated whether the external limiting membrane (ELM) and ellipsoid zone (EZ) were continuous or defect after surgery, both ELM and EZ were defect (B), ELM was continuous and EZ was defect (C), both ELM and EZ were continuous (D).

All patients underwent comprehensive ophthalmologic examinations, including stereopsis, BCVA, and foveal microstructure using OCT at baseline and 3, 6, and 12 months after surgery. Stereopsis was evaluated using the Titmus Stereo Test (TST) and the TNO stereo test (TNO) at the viewing distance of 40 cm with appropriate spectacle correction. Patients’ responses were checked by inverting the stereo target and asking if the target appeared in front of or behind the page to ensure that the patients were not using monocular clues in the TST. Microstructural imaging analysis of the fovea was performed using spectral-domain OCT (SD-OCT) (Cirrus high definition OCT; Carl Zeiss, Dublin, CA, USA). Five-line raster scans in horizontal of each eye were performed by using commercial analysis software (Cirrus version 3.0 analysis software; Carl Zeiss) with a signal intensity of ≥ 7 (maximum of 10) and were defined as suitable images. Four parameters, including base MH diameters, minimum MH diameters at baseline, and the ELM and ellipsoid zone (EZ) status after surgery, were evaluated using OCT. The MH’s base and the minimum diameters were measured manually using a caliper. The base diameter was measured at the base of the MH just above the retinal pigment epithelium, and the minimum MH diameter was measured at the narrowest part of the MH. After surgery, ELM and EZ recovery were evaluated in defect and continuous (Fig 1). Two masked investigators (T.M. and Y.S.) evaluated the SD-OCT images. All ophthalmic examinations were performed by skilled orthoptists.

We divided MH patients into two groups: patients who had undergone vitrectomy with complete ILM peeling (Peeling group) and with the inverted ILM flap technique (Inverted group). The results of the TST and TNO were expressed as seconds of arc, and we converted these values to logarithms for statistical assessment. BCVA was measured with the Landolt chart and expressed as the logarithm of the minimum angle of resolution (logMAR). The mean and standard deviation were calculated for the patient’s age, TST score, TNO score, BCVA, and MH’s base and minimum diameters. The Mann-Whitney *U* test was used to compare age, TST, TNO scores, BCVA, and MH parameters between the Peeling and Inverted groups. The chi-square test was used to check for sex-related between-group differences. We compared the stage of MH between the Peeling and Inverted groups using Pearson’s chi-squared test. Fisher’s exact probability test was used to compare the MH closure rate, the ELM, and EZ status between the ILM peeling and the inverted ILM flap groups. The TST, TNO, and BCVA were analyzed using a repeated-measures analysis of variance to assess the time course of the changes. If significant differences were found, a Bonferroni test was performed. Unpaired t-test was carried out in the analysis to compare the TST, the TNO score, and BCVA at baseline, 3, 6, and 12 months after surgery between the two groups. Single regression analysis was used to examine the correlation between the visual acuity of fellow eye and stereopsis. The same analysis was used for BCVA after surgery and stereopsis. The analysis of stereopsis outcomes was performed except for the case that failed to close after the surgery. All statistical analyses were performed by SPSS software 25.0 (SPSS, Chicago, IL, USA). A *P*-value <0.05 was considered statistically significant.

## Results

The study showed that 41 eyes of 41 patients (18 men, 23 women) had undergone vitrectomy with the ILM peeling (Peeling group), and 25 eyes of 25 patients (11 men, 14 women) had undergone vitrectomy with the inverted ILM flap technique (Inverted group). Clinical characteristics of patients with MH at baseline were summarized in Table 1. The TST values at baseline were 2.7 ± 0.5 and 2.9 ± 0.5 in the Peeling and Inverted groups, respectively. The TNO values at baseline were 2.7 ± 0.5 and 2.7 ± 0.4 in the Peeling and Inverted groups, respectively. No statistical difference was observed in stereopsis and MH size between the Peeling and Inverted groups.

**Table 1:** Clinical characteristics of patients underwent vitrectomy with the ILM peeling or with the inverted ILM flap technique for MH at baseline.

	Peeling group	Inverted group	P value
Number of eyes	41	25	
Age (years)	65.1 ± 5.6	66.1 ± 6.2	0.495
Gender (men / women)	18 / 23	11 /14	0.993
Stereopsis			
Titmus Stereo Test (log)	2.7 ± 0.5	2.9 ± 0.5	0.110
TNO stereo test (log)	2.7 ± 0.5	2.7 ± 0.4	0.838
Best-corrected visual acuity (logMAR)	0.74 ± 0.33	0.72 ± 0.33	0.107
Stage of MH (stage 2/ 3/ 4)	8/ 22/ 11	6/ 7/ 12	0.102
Mnimum diameter of MH (μm)	396 ± 201	415 ± 173	0.639
Base diameter of MH (μm)	806 ± 293	821 ± 234	0.858

Values are presented as the mean ± standard deviation.

The Mann-Whitney U test, the chi-square test and Pearson’s chi-squared test was used.

logMAR: logarithm of the minimum angle of resolution, ILM: internal limiting membrane, Inverted group: vitrectomy with inverted ILM flap technique, Peeling group: vitrectomy with ILM complete ILM peeling, MH: macular hole.

No complications such as retinal detachment, proliferative vitreoretinopathy, secondary epiretinal membrane, and endophthalmitis after MH surgery were observed. After 12 months of follow-up, OCT showed MH closure in 40 (97.6%) cases in the Peeling group and 25 (100%) cases in the Inverted group, with no significant difference between the two groups.

**Fig 2:**
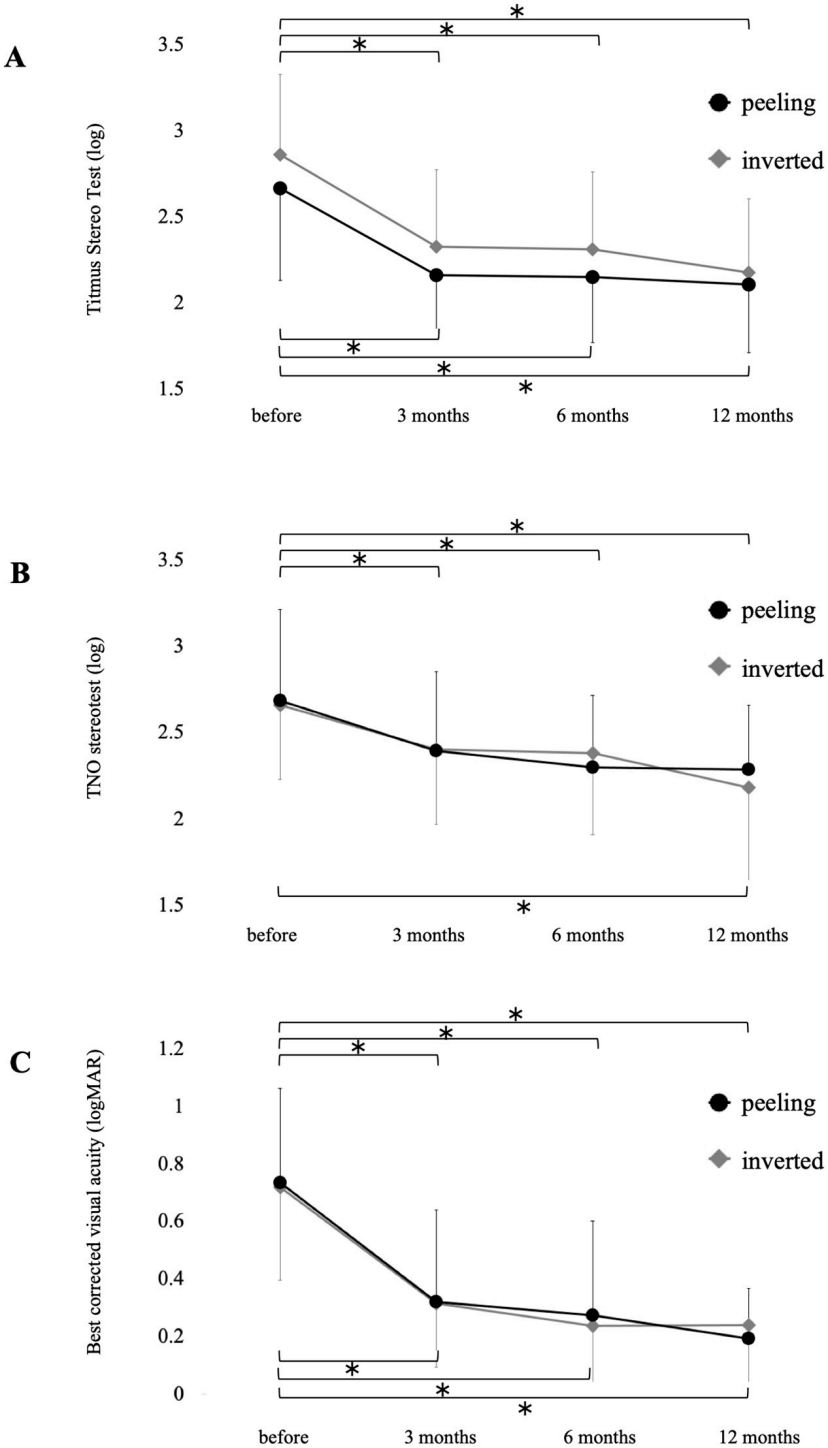
Time course of changes in stereopsis and best-corrected visual acuity in patients with macular hole before and after surgery with the internal limiting membrane (ILM) peeling or the inverted ILM flap technique for 12 months. **A**: The Titmus Stereo Test values. **B**: The TNO stereo test values. **C**: The best-corrected visual acuity. *One-way repeated measures analysis of variance with a Bonferroni test.

Fig 2 shows the time course of change of stereopsis and BCVA in the Peeling and Inverted groups. The TST value improved significantly at 3, 6, 12 months after surgery in the Peeling and Inverted groups. At 12 months postoperatively, the TST score was 2.1 ± 0.4 in the Peeling and 2.2 ± 0.4 in the Inverted groups. Furthermore, the two groups had no significant difference in TST value throughout the observation period (Fig 2A). The TNO value also improved significantly at 3, 6, and 12 months after surgery in the Peeling group, whereas in the Inverted group, the TNO value improved significantly only 12 months after surgery. At 12 months postoperatively, the TNO score was 2.3 ± 0.4 in the Peeling and 2.2 ± 0.5 in the Inverted groups. No significant difference was found in the TNO value between both groups throughout the observation period (Fig 2B). Both surgical procedures improved BCVA, and there was no significant difference in BCVA between the two groups during the follow-up period (Fig 2C). The visual acuity of the fellow eye was not correlated with the TST and TNO. The BCVA of the eyes after surgery were correlated with the TST, but not TNO.

**Fig 3:**
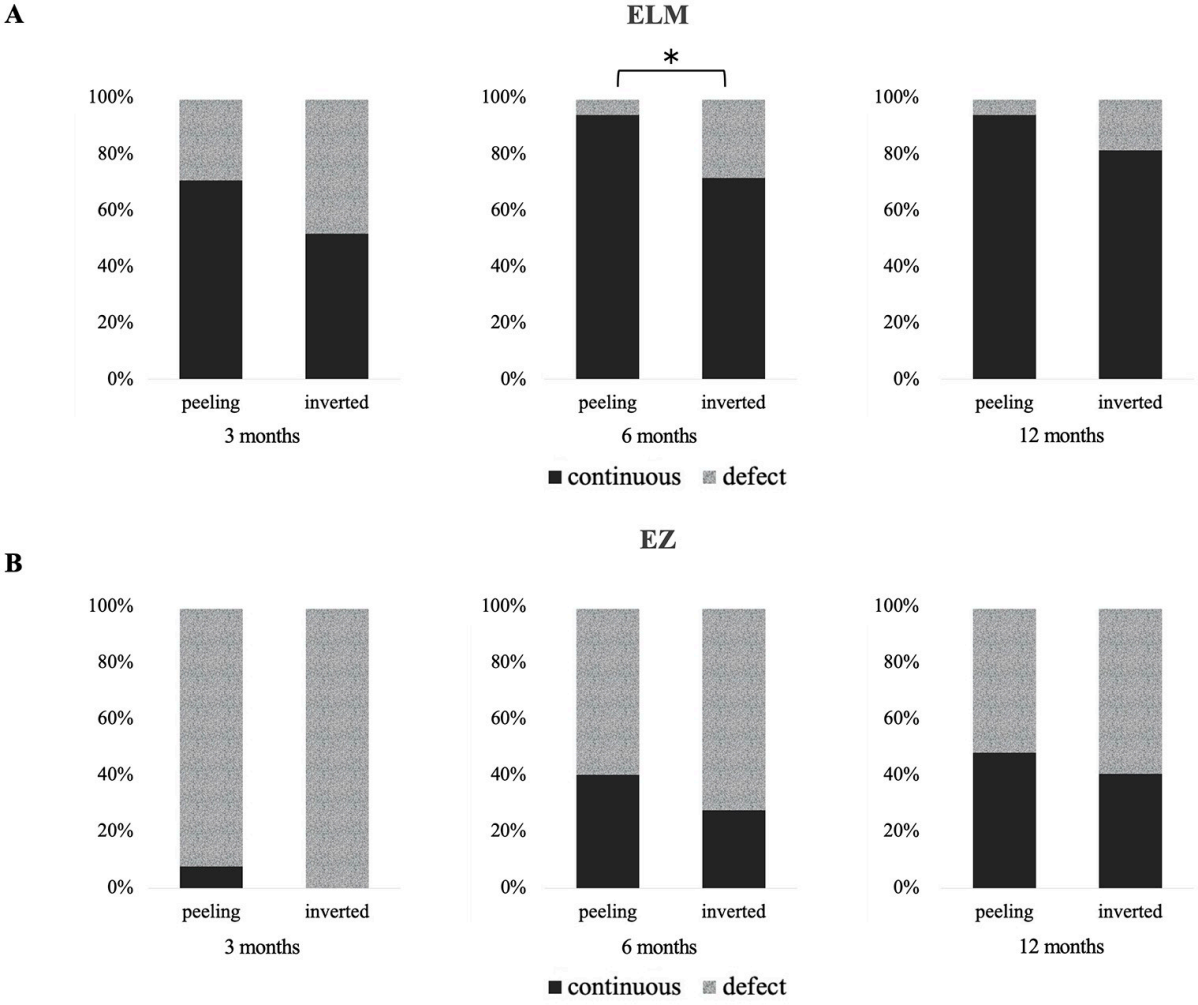
Status of the external limiting membrane (ELM) and ellipsoid zone (EZ) in the internal limiting membrane (ILM) peeling group and the inverted ILM flap group at 3, 6, and 12 months after surgery. **A**: Ratio of continuous restoration of ELM was higher in the Peeling group than in the Inverted group during the follow-up period. The status for the ELM at 6 months after surgery in the Peeling group was better than that in the Inverted flap group. **B**: Ratio of continuous restoration of EZ was higher in the Peeling group than in the Inverted group during the follow-up period. There was no statistical difference in the status of EZ between the Peeling and the Inverted groups at 3, 6, and 12 months after surgery. *Fisher’s exact probability test.

The retinal outer layer status after MH surgery is shown in Fig 3. Both surgical technique groups showed gradual recovery. The ratio of continuous restoration of ELM was higher in the Peeling group than in the Inverted group in the observation period. The ELM score at 6 months after surgery in the Peeling group was higher than that in the Inverted group (Fig 3A). The ratio of continuous restoration of EZ was higher in the Peeling group than in the Inverted group in the observation period. However, there was no statistical difference in EZ status between the Peeling and Inverted groups at 3, 6, and 12 months after surgery (Fig 3B).

Subgroup analyses were performed for MH with minimum diameters larger than 400 μm. Nineteen eyes were assigned to the Peeling group and 12 to the Inverted group. The anatomical success rates achieved in the Peeling and Inverted groups were 94.7% and 100%, respectively (p = 0.419). No significant difference was observed in stereopsis and BCVA between the two groups during the follow-up period. The same analyses were performed for MH with minimum diameters smaller than 400 μm. No significant difference was also observed in stereopsis and BCVA between the Peeling and the Inverted groups during the follow-up period.

## Discussion

There are several types of vitrectomy for MH, including simple vitrectomy, ILM peeling, and the inverted ILM flap technique, and functional and anatomical have been improved [11, 17–21]. Baumann reported that vitrectomy with the ILM peeling and the inverted ILM flap technique showed significant improvements in patients’ preoperative to postoperative BCVA at 3 months. However, there was no significant difference between the two groups [23]. A previous study reported that the ELM postoperative recovery rates in the inverted ILM flap group were lower than those in the ILM peeling group [25]. However, there are no reports of stereopsis between the two techniques. We compared the stereopsis of the vitrectomy using the ILM peeling technique with that of the inverted ILM flap, and both surgical techniques improved the TST and TNO scores. There was no significant difference in the stereopsis score between the two groups throughout the follow-up period. BCVA was improved by the ILM peeling and the inverted ILM flap, and there was no significant difference in BCVA between both groups. Thus, either surgical procedure for patients with MH was useful for visual functions such as stereopsis and BCVA.

The ELM status at 6 months after surgery in the Peeling group was better than that in the Inverted group, but the ELM status at 12 months after surgery was the same for both groups in our study. Iwasaki and associates reported that the ELM recovery period in the Inverted group was significantly longer than that in the Peeling group [25]. Iuliano L. et al. the surgical repair of small full-thickness MH with the inverted ILM flap technique seem to delay the foveal structural repair and to gain an inferior foveal sensitivity compared to the standard technique [26]. When performing an inverted ILM flap, the physical obstruction created by the ILM flap may delay the recovery of the outer retinal layers. Vitrectomy with ILM peeling for MH might be expected to recover the outer retina more quickly than that with the inverted ILM flap technique, while there was no significant difference between the two groups for a year.

Subgroup analysis of this study suggested that the Peeling and Inverted groups had similar anatomic success rates in patients with large MH, defined as minimum diameters of 400 μm or greater. Several studies exist on MH closure rates in patients with MH by vitrectomy with ILM peelings and reverse ILM flap technique. Rizzo S. et al. reported that vitrectomy associated with the inverted ILM flap technique seems to be a more effective surgery for idiopathic and myopic large MHs than only ILM peeling, improving both functional and anatomical outcomes [17]. Yamashita and associates demonstrated that in extra-large MHs, the closure rate was 88.4% by the conventional ILM peeling and 100% by the inverted ILM flap technique, and the difference between the two methods was not significant [19]. In a previous study, although the difference between the two methods was not significant, the inverted ILM flap method was successful in 100% of the cases. Consequently, vitrectomy with the inverted ILM flap might be a better surgical technique for large MH from an anatomic success viewpoint.

Taking into consideration, the two surgical techniques for MH, including the ILM peeling and the inverted ILM flap, improved not only BCVA but also stereopsis. This is the first report to compare vitrectomy with the ILM peeling and the inverted ILM flap technique with stereopsis in patients with MH. Vitrectomy with the inverted ILM flap tended to have a better MH closure rate than the ILM peeling. A previous study reported that the ILM inverted flap surgical procedure promoted faster recovery of BCVA and outer retinal layers and was more protective against postoperative foveal thinning and displacement than the ILM peeling [27]. Chou HD. et al. concluded that ILM flaps were associated with better visual outcomes up to 6 months postoperatively and should be considered in small MHs [28]. On the other hand, Ventre L. et al. argued that Inverted flap technique has disadvantages compared with conventional peeling for the treatment of small-to-medium idiopathic MHs [29]. In this study, vitrectomy with the ILM peeling may recover the ELM more quickly than that with the inverted ILM flap technique, while there was no significant difference between the two groups for 12 months after surgery. In addition, there was no significant difference in BCVA and stereopsis, which was the most advanced visual function in the Peeling and Inverted groups. Further studies are needed to assist the vitreoretinal surgeon in determining the surgical approach for MH and in explaining the procedure to the patient.

There were several limitations of this study. First, this was a retrospective study. Second, the sample size was relatively small. Prospective studies with larger sample sizes are required. Third, there was only one surgical procedure of the inverted ILM flap, which was performed according to the ordinal method of report by Michalewska et al. [11]. Various surgical techniques for MH, including hemi-temporal ILM peeling [30–33] and superior inverted ILM flap [34, 35], have been reported. Forth, not only the continuous or defect of ELM and EZ, but also more detailed data such as length should be considered in the evaluation.

In conclusion, we have compared stereopsis, anatomical results, and the foveal microstructure of the conventional ILM peeling versus the inverted ILM flap technique for treating MH. No difference was observed in stereopsis after surgery between vitrectomy with the ILM peeling and inverted ILM flap. The microstructure of the retina improved more quickly in the ILM peeling than in the inverted ILM flap. However, there was no significant difference between the two groups for 1 year after surgery.

## Supporting information

S1 Dataset Information including sex, age, MH size, stereopsis and BCVA of patients. (XLSX)
